# Zebrafish: An Attractive Model to Study *Staphylococcus aureus* Infection and Its Use as a Drug Discovery Tool

**DOI:** 10.3390/ph14060594

**Published:** 2021-06-21

**Authors:** Sari Rasheed, Franziska Fries, Rolf Müller, Jennifer Herrmann

**Affiliations:** 1 Helmholtz Institute for Pharmaceutical Research Saarland (HIPS), Helmholtz Centre for Infection Research, Saarland University Campus, 66123 Saarbrücken, Germany; Sari.Rasheed@helmholtz-hips.de (S.R.); Franziska.Fries@helmholtz-hips.de (F.F.); Rolf.Mueller@helmholtz-hips.de (R.M.); 2 German Centre for Infection Research (DZIF), Partner Site Hannover–Braunschweig, 38124 Braunschweig, Germany; 3 Department of Pharmacy, Saarland University, 66123 Saarbrücken, Germany

**Keywords:** zebrafish, *Staphylococcus aureus*, non-mammalian models, invertebrates, bacterial infection, drug discovery

## Abstract

Non-mammalian in vivo disease models are particularly popular in early drug discovery. Zebrafish (*Danio rerio)* is an attractive vertebrate model, the success of which is driven by several advantages, such as the optical transparency of larvae, the small and completely sequenced genome, the small size of embryos and larvae enabling high-throughput screening, and low costs. In this review, we highlight zebrafish models of *Staphyloccoccus aureus* infection, which are used in drug discovery and for studying disease pathogenesis and virulence. Further, these infection models are discussed in the context of other relevant zebrafish models for pharmacological and toxicological studies as part of early drug profiling. In addition, we examine key differences to commonly applied models of *S.* *aureus* infection based on invertebrate organisms, and we compare their frequency of use in academic research covering the period of January 2011 to January 2021.

## 1. Introduction

*Staphylococcus aureus* is a Gram-positive opportunistic pathogen responsible for nosocomial and community-acquired infections characterized by high mortality rates. *S. aureus* comprises a multitude of virulence factors, including adhesins, immune evasion factors and various toxins, allowing the bacteria to adhere to cells and other surfaces, invade the host tissue and evade both the innate and acquired immune system [1]. An infection with *S. aureus* can cause several diseases that vary from minor superficial lesions to specific syndromes, such as toxic shock syndrome (TSS) and scalded skin syndrome (SSSS), to life-threatening conditions such as endocarditis, osteomyelitis, pneumonia and bacteremia. Moreover, *S. aureus* forms biofilms, making this pathogen one of the leading causes of biomaterial-associated infections (BAI) [1,2]. In 1959, methicillin was introduced as the first semisynthetic penicillinase-resistant β-lactam antibiotic and, only two years later, the first methicillin-resistant strains were found among *S. aureus* isolates [3,4]. Until today, infections caused by methicillin-resistant *S. aureus* (MRSA) remain one of the greatest threats to human health. For treatment of MRSA infections, vancomycin—an antibiotic that disrupts cell wall biosynthesis by interacting with the terminal d-Ala-d-Ala motif of the precursor lipid II—belongs to the first-line drugs [5,6]. However, in recent years, there has been an increasing rate of vancomycin-intermediate (minimum inhibitory concentration, MIC = 4–8 μg/mL) and vancomycin-resistant *S. aureus* (MIC ≥ 16 μg/mL) [5]. *S. aureus*, including methicillin-resistant, vancomycin-intermediate (VISA) and vancomycin-resistant *S. aureus* (VRSA), is, according to the World Health Organization (WHO), a high priority pathogen for which new antibiotics are urgently needed [7]. In case of infections caused by VRSA, the use of second-line drugs such as daptomycin, linezolid and tigecycline is recommended [5]. However, resistance to these last resort antibiotics has also emerged in the recent past [8]. This rapid and wide spread of resistant mutants urges the discovery of new antimicrobials with a novel mode of action. In order to identify new targets, it is vital to understand the pathogenesis of *S. aureus* on a molecular basis and to identify virulence factors that are essential to the occurrence of disease.

Animals, and rodents in particular, are widely used models to study infectious diseases as they represent important tools for the preclinical evaluation of new antimicrobials [9]. Mammalian models exist for a variety of pathophysiologies generated by staphylococci [10,11,12,13,14]; however, their usage comes along with ethical issues, as well as low throughput and high costs. Non-mammals, including primary invertebrates and vertebrate zebrafish larvae, have been used more frequently in recent years as excellent alternatives to higher animal models in order to explore disease pathogenesis, to study host–pathogen interactions, and to assess the efficacy and safety profiles of novel anti-infective agents.

This review provides an overview on zebrafish models of *S. aureus* infection, highlights possibilities and advantages of this vertebrate model and points out the key differences to commonly used invertebrate models. The zebrafish models of infection are also discussed in a broader context, highlighting their use in early drug discovery in conjunction with other relevant models to assess safety profiles and pharmaceutical properties of new antibacterial agents.

## 2. Non-Mammalian Models in Pharmaceutical Research

**Figure 1:**
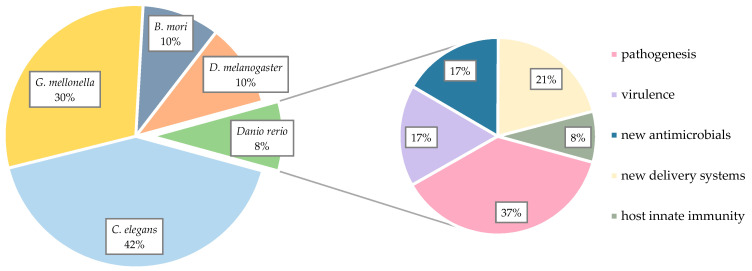
Percentage of different non-mammalian in vivo models used for *Staphylococcus aureus* infection (**left**) and applications of zebrafish models to study *S. aureus* infection (right) over the last decade. A PubMed database search was performed to identify studies using invertebrates (*Galleria mellonella*, *Caenorhabditis elegans*, *Drosophila melanogaster* and *Bombyx mori*) and zebrafish embryos/larvae for *S. aureus* infection models. Studies from January 2011 through January 2021 were included and percentages were calculated based on the total number of publications (*n* = 282) in this period (see SI). *G. mellonella* and *C. elegans* are the most used models for studying different aspects of *S. aureus* infection and for assessing the in vivo efficacy of new potential antibiotics. Although zebrafish larvae are prominent models for studies of infectious disease and have been used for a broad range of other microorganisms, there are only relatively few literature reports on the use of this model to study *S. aureus* infection. The fields of applications of zebrafish models of *S. aureus* infection are depicted on the right. Most studies were performed with the intention of analyzing the pathogenesis and the virulence of different strains. Moreover, zebrafish larvae seem to be a promising platform for studying the in vivo efficacy of new anti-staphylococcal agents and new delivery systems/routes for already known antibiotics.

The use of non-mammalian models for in vivo studies has increased drastically over the last two decades. Their simplicity and the high conservation of many aspects of the innate immune system between invertebrates, higher animals and even humans [15,16] make them attractive tools to overcome logistical, ethical and financial limitations of classical model organisms such as mice, rats, guinea pigs, rabbits or non-human primates. In order to analyze the most frequently used non-mammalian models for *S. aureus* infection in the literature, we performed a PubMed database search and we included studies from January 2011 through January 2021 (Figure 1; search details can be found in the SI). *Caenorhabditis elegans* (roundworm/nematode), *Galleria mellonella* (waxworm), *Drosophila melanogaster* (fruit fly) and *Bombyx mori* (silkworm) represent common invertebrate organisms used for studying different aspects associated with staphylococcal infection. Studies using zebrafish (*Danio rerio)* were included when experiments were performed with embryos or larvae within the first 120 h post-fertilization (hpf) since experiments using zebrafish in these early life stages are not considered as animal experiments according to the EU Directive 2010/63/EU [17].

As displayed in Figure 1, most *S. aureus* infection models published over the last decade were performed with *C. elegans* as the host of choice, followed by the waxworm, *G. mellonella*. In fact, a clear trend towards invertebrate models can be observed and zebrafish larvae, as host organisms, are thus far underrepresented. This could be due to several reasons. Firstly, there is the financial aspect; compared to invertebrates, zebrafish come along with higher costs for housing and husbandry, yet these costs are still much lower than with higher animals [18]. In addition, some injection techniques required for the infection of zebrafish are quite laborious and, depending on the purpose of the study, it might be more convenient to choose a less complex model organism. Nonetheless, zebrafish as vertebrates offer some important benefits, which are highlighted below. The majority of publications using zebrafish as model hosts focused on the study of *S. aureus* pathogenesis and virulence mechanisms of different strains. In addition, zebrafish larvae represent a popular platform for studying the in vivo efficacy of novel anti-staphylococcal agents and delivery systems/routes for already known antibiotics.

*Caenorhabditis elegans* presents a number of advantages that makes it an attractive model in infection research. This includes its small and transparent body, small sequenced genome (100 Mb), low cost, ease of handling, short life cycle and self-fertilization. Furthermore, *C. elegans* feeds on bacteria, which facilitates the infection route with bacterial pathogens [19]. On the other hand, the nematode lacks an adaptive immune system and the worm cannot be incubated at 37 °C, which is the optimal temperature for the expression of some virulence factors by a number of bacterial pathogens [20].

*Galleria mellonella* larvae also represent an attractive model to study fungal, bacterial and viral pathogens as well as to assess virulence and toxicity. Moreover, they are a popular platform for the screening of antimicrobial drugs. They are cheap, easy to maintain, and do not require ethical approval. Unlike *C. elegans*, *G. mellonella* larvae can be maintained at 37 °C [21]. Although the *G. mellonella* model has become popular, infection models are not well established, as they are for other models such as *C. elegans* and *D. melanogaster* [22].

*Drosophila melanogaster* is a well-known and historic model that has been used in research for around 100 years. The fruit fly has been widely used as a model organism in genetics, biochemistry, developmental biology, cancer, infectious diseases, neurodegenerative diseases, inflammation and metabolic disorders, as well as in toxicological studies [23,24]. The fruit fly is easy and inexpensive to maintain in large quantities, genetic manipulation is rapid and cheap, it has a short generation time and short lifespan, and its genome is fully sequenced. Furthermore, 75% of human disease-causing genes have a functional homolog in *D. melanogaster* [25]. On the other hand, a number of drawbacks hinder the use of this model, such as the absence of an adaptive immune system and the significant difference in brain anatomy as well as cardiovascular and respiratory systems compared to vertebrates [26].

*Bombyx mori* is another interesting and unique model that is frequently used in infection research. In addition to their small size, ease of handling and sequenced genome, their sensitivity to chemical compounds such as pesticides, drugs, and heavy metals make them an important model in toxicological screens and drug discovery approaches [27]. A major disadvantage is that the silkworm larvae display a rather low homology in terms of disease-related genes and genetic disorders compared to humans, which makes this model not suitable to study, for example, neurological disorders [28].

Despite numerous advantages of the most commonly used invertebrates mentioned above, such models lack organ systems that are involved in human disease pathogenesis, which limits their usage in human disease modeling. As a result, zebrafish (*Danio rerio*) has been introduced as an attractive vertebrate model. The usage of zebrafish in research initially started in the 1930s in developmental studies; however, it has recently been used as a model of choice in several human diseases [29]. The most important advantages of the zebrafish model include the optical transparency of embryos and larvae, the high homology with respect to human disease-related genes based on a completely sequenced genome, the small size of embryos and larvae and their ease of handling, and low cost [30]. Importantly, unlike *C. elegans* and *D. melanogaster*, zebrafish have an adaptive immune system [31].

Zebrafish are susceptible to bacterial (including mycobacterial), protozoal, and viral infections, allowing the development of various infection models, including pathogens that are usually not considered as natural pathogens of the fish, such as *S. aureus*. In the pipeline of drug discovery, zebrafish larvae are extensively used to study drug efficacy and to assess toxicity. The readouts for such approaches include survival studies or visual phenotypic assessment. Furthermore, several transgenic zebrafish lines with fluorescent reporter genes have been generated, facilitating the visualization of pathogenic processes [32,33,34,35].

## 3. Zebrafish Larvae Models of *S. aureus* Infections

Due to its many advantages, the zebrafish has emerged as an excellent vertebrate model organism used in various fields, including developmental biology, immunology, toxicology and pharmaceutical drug discovery. As a jawed vertebrate, the zebrafish has developed both innate and adaptive immune systems [36]. The adaptive immune system is fully mature by 4–6 weeks post-fertilization [37]; thus, at earlier developmental stages, the zebrafish larvae are protected against infection only by the innate immune system, enabling the investigation of innate defense mechanisms separated from adaptive immunity. Innate immune responses in zebrafish comprise innate immune cells, such as macrophages and granulocytes [38], pattern recognition receptors, including Toll-like receptors (TLRs) and NOD-like receptors (NLRs) [39], and a highly developed complement system [40]. Despite the evolutionary distance, comparison of the zebrafish to the human reference genome revealed high homology; in fact, over 70% of human genes have one or more orthologues in zebrafish. Among human disease-related genes, the homology is even higher (82%) [41], which makes the vertebrate appropriate for modelling human diseases. Another major advantage of zebrafish is the optical transparency of embryos and larvae. Along with the availability of transgenic zebrafish lines expressing fluorescent proteins in different cell types and tissues, this facilitates the investigation of host–pathogen interactions in real time and in vivo [31] without the need for invasive measures.

**Figure 2:**
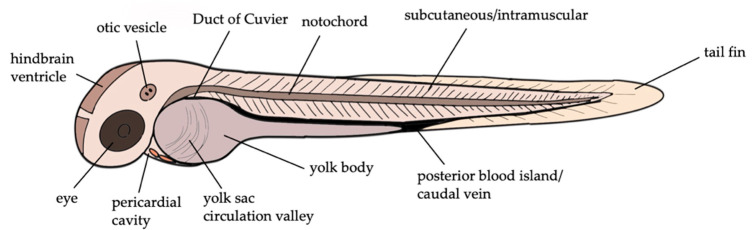
Injection sites of zebrafish larvae. Injection into the yolk sac circulation valley, Duct of Cuvier and the caudal vein leads to systemic infection, whereas injection into the yolk body, pericardial cavity, eye, otic vesicle and hindbrain ventricle results in local growth of bacteria. The figure was generated using SketchBook version 8.7.1.

As depicted in Figure 2, zebrafish larvae provide various injection sites. The development of a systemic bacterial infection can be achieved via microinjection into the yolk sac circulation valley, the Duct of Cuvier and the caudal vein/blood island, whereas local growth of bacteria occurs when injected into the yolk body or a body cavity such as the otic vesicle or the fourth hindbrain ventricle [42]. The choice of an appropriate route of infection may also depend on the respective research question; for example, the pericardial cavity provides a potential approach for pericarditis, the hindbrain can be used to study infection of the central nervous system (CNS), a systemic route of infection offers a suitable bacteremia model, and other compartments such as the tail fin and the muscle permit investigations of chemotaxis of immune cells.

All research papers published between January 2011 and January 2021 describing different zebrafish larvae models of *S. aureus* infection are summarized in Table 1; Table 2. Herein, we highlight some of the most interesting findings related to disease biology (this Chapter, Table 1) and drug discovery (Chapter 4, Table 2).

Li et al. (2012) have previously shown that the choice of injection site has a great impact on the level of resistance towards the development of an infection with *S. aureus*. Challenging zebrafish larvae at 36 h post fertilization (hpf) with rising doses of the pathogen via different injection sites revealed that injection of *S. aureus* into local cavities such as the pericardial cavity, the eye and the hindbrain induce a strong host defense, resulting in low infection rates. In contrast, the use of injection sites leading to systemic *S. aureus* infection results in significantly lower survival rates. The yolk body represents the most susceptible injection site to staphylococcal infection, as almost every dose will eventually lead to 100% mortality of zebrafish larvae. This phenomenon can be explained by the high nutrition supply and the absence of immune cells providing the bacteria with good conditions to proliferate [43]. By using transgenic zebrafish lines with fluorescently labeled phagocytes, Li et al. visualized host–pathogen interactions and confirmed the role of innate immune cells, mainly macrophages and neutrophils, in the primary resistance of zebrafish larvae against *S. aureus*, as already reported by Prajsnar et al. (2008) [32,43].

McVicker et al. (2014) have reported the usage of a zebrafish larvae model to study the in vivo effect of administering low levels of antibiotics on the bacterial population dynamics of *S. aureus*. In their approach, they have systemically infected zebrafish embryos at 30 hpf with 1.5 × 10^3^ colony-forming units (CFU) of a mixture of three *S. aureus* antibiotic-resistant strains (erythromycin/lincomycin-resistant, kanamycin-resistant and tetracycline-resistant). Upon culturing homogenized whole zebrafish embryos and enumeration of bacteria, they found that there was no preference for the growth of any particular strain over the other, and that the clonal population expansion is random. Furthermore, these authors have studied the clonal expansion of bacteria during mixed-strain infections of drug-resistant mutant strains and sensitive strains after treatment with low antibiotic concentrations. For this approach, they have treated (by immersion in fish water) infected zebrafish larvae with sub-curative tetracycline concentrations. Their results suggested that sub-curative drug treatment was able to produce a statistically significant shift in strain ratios with a preference towards the pre-existing resistant subpopulation. It is useful to mention that, in their research paper, they have studied the same approach not only in zebrafish but also in mice, and they found similar results [47].

Ulhuq et al. (2020) have used the zebrafish hindbrain as a local site of infection with *S. aureus*. For their approach, they have infected zebrafish larvae 3 days post fertilization (dpf) with 2 × 10^4^ CFU in order to investigate the effect of type VII protein secretion system (T7SS) in bacterial replication and its role in intraspecies competition. The in vivo virulence of different mutant *S. aureus* strains was assessed via survival studies, recovery of colonies and investigation of the cytokine response upon infection by quantification of interleukin (IL)-8 and IL-1β using quantitative reverse transcription PCR (qRT-PCR). Moreover, they studied intraspecies competition mediated by T7SS-secreted toxins by co-infecting larvae with an attacker and a target strain, whereby the target strain represents a mutant strain deprived of immunity proteins for the toxins EsaD and TspA. It could be shown that the attacker strain is able to kill its target in vivo in a T7SS-dependent fashion, as reflected in reduced bacterial counts of the target strain upon plating of homogenized larvae [34].

In a recent research paper, Bhuiyan et al. (2021) have used a zebrafish infection model to study the host innate immune responses after infection with daptomycin-resistant and susceptible *S. aureus* strains. For this, they have infected zebrafish larvae 48 hpf via the Duct of Cuvier with 1 × 10^3^ CFU. Furthermore, they have inhibited hepcidin—a major zebrafish antimicrobial peptide—either chemically by incubating 30 hpf embryos in fish water with dorsomorphin along the course of the experiment, or genetically by injecting antisense morpholino oligomers (MO) directed at *hamp* (*hamp*ATG MO) in the yolk of one-cell stage embryos. They have reported that infection with daptomycin-susceptible *S. aureus* caused greater mortality when hepcidin was inhibited, demonstrating the importance of cationic antimicrobial peptides (CAMPs) in controlling *S. aureus* infection. On the other hand, infection with daptomycin-resistant strains was not affected by the presence or absence of zebrafish hepcidin, most likely due to cross-resistance of daptomycin and CAMPs. In this study, zebrafish larvae presented a major in vivo insight into the pathogenicity of daptomycin-resistant *S. aureus* strains [54].

In conclusion, zebrafish larvae are becoming an important in vivo model in *S. aureus* infection research, and the model is already used to study, e.g., bacterial virulence, disease pathogenesis, host–pathogen interactions, and host immune responses. The major advantages of the zebrafish larvae model in this context are the physiological and morphological similarity to mammals, the broad availability of genomic tools, the ease of performing infections in different sites and at different developmental stages, and the ability to visualize bacterial infection in real-time and to study disease pathogenesis by fluorescence microscopy.

## 4. Zebrafish Larvae in Drug Discovery

Regulatory directives in several countries have implemented the 3R principle for animal protection and for minimizing their pain and distress in biomedical research. This principle aims to avoid animal experiments (Replacement), to limit the number of animals (Reduction), and to limit their suffering (Refinement) [55,56]. In drug discovery research, an enormous number of discovered bioactive compounds are tested for their safety, bioavailability and efficacy in animal models, and a high percentage of such compounds do not progress due to their toxicity profiles or unfavorable pharmacokinetic properties. Given the 3R principle and, at the same time, the need to assess the pharmaceutical properties of early-stage compounds, there is a high need to improve preclinical testing. Using zebrafish embryos and larvae represents an alternative approach for high-throughput in vivo drug screening.

Several screens have been assessed in zebrafish embryos, and these include, but are not limited to, cardiotoxicity, hepatotoxicity, embryogenesis, carcinogenesis, behavioral studies, metabolic and nutritional diseases, endocrine diseases, inflammation and wound healing, and neurological diseases [31]. Zebrafish larvae also play an important and unique role in drug screening, target identification, pharmacological and toxicological studies [57]. The vertebrate model presents an advantage to assess phenotype-based screens for a wide range of compounds in the drug discovery pipeline.

Table 2 summarizes all published reports between January 2011 and January 2021 on the use of zebrafish larvae in *S. aureus* drug discovery. In the following, some research highlights are described in more detail.

Drug efficacy studies and in vivo anti-infection assays using zebrafish larvae have been widely applied and new models are continuously developed. In a recent paper published by Jabila Mary et al. (2021), the antibacterial effect of Kalafungin isolated from a marine sponge-derived *Streptomyces* sp. was investigated. For this approach, an infection model was established via microinjection of green-fluorescent protein (GFP)-tagged *S. aureus* into the caudal vein of zebrafish larvae. The infected larvae were then treated though immersion in fish water with 0.5-, 1- and 2-fold MIC (minimum inhibitory concentration) of Kalafungin. Treatment with sub-MIC of Kalafungin resulted in a bacteriostatic effect lasting up to 36 hpt (hours post treatment), whereas treatment with 1- and 2-fold MIC completely cleared the infection within 12 hpt. Another approach was the infection of larvae by bath immersion with 10^4^ CFU/mL. Fluorescence microscopy revealed accumulation of bacteria in the Duct of Cuvier, the cardiac region and the yolk sac, proving that *S. aureus* is able to infect the larvae without the need for microinjection. Kalafungin treatment performed at 12 hpi (hours post infection) with 1- and 2-fold MIC showed that the Kalafungin is also efficient in clearing infection induced via bath immersion [60].

Sovari et al. (2020) have reported the use of zebrafish larvae for in vivo toxicity assessment and therapeutic efficacy studies in a zebrafish model of MRSA infection for a number of rhenium (Re) complexes. A systemic infection was initiated through microinjection into the caudal vein of 30 hpf embryos. The infected embryos were treated with 0.5-, 1- and 2-fold MIC doses of the selected Re complexes. The therapeutic efficacy of the complexes was obvious at 24 hpt, as shown by higher survival rates of the treated group compared to the untreated group, the absence of necrotic lesions (abscesses) or pericardial edema. Moreover, most Re complexes showed an enhanced in vivo antibacterial effect compared to linezolid after 4-days treatment. The authors further reported that all rescued larvae developed normally post treatment without toxic side effects. In this study, the in vivo activity and safety of Re complexes could be nicely demonstrated in zebrafish larvae without the need of a higher animal model [58].

*S. aureus* is a facultative intracellular pathogen since it can survive in host cells, thus escaping detection by professional phagocytes [67]. Such characteristic presents an additional obstacle in treating *S. aureus* infections due to the low intracellular efficacy of many antibiotics. Therefore, there is an urgent need for the development of innovative delivery systems to improve the therapeutic efficacy of antibiotics. In this context, zebrafish larvae present an attractive model to assess and validate the in vivo efficacy of drug-delivery systems. Zhang et al. (2018) have explored the usage of gelatin nanospheres as carriers of vancomycin into macrophages of zebrafish larvae. The distribution and internalization of fluorescently labeled gelatin nanospheres were assessed by injecting a volume of ~3 nL systemically via the Duct of Cuvier or locally into the tail muscle tissue of 3 dpf larvae. The same approach was followed to study the interaction between zebrafish macrophages and vancomycin-loaded gelatin nanospheres compared to free vancomycin in a transgenic zebrafish line that expresses mCherry fluorescent protein in their macrophages. Furthermore, they have investigated the effect of systemic versus local delivery of gelatin-coated vancomycin on survival of *S. aureus*-infected zebrafish larvae. They found out that gelatin nanospheres were internalized by zebrafish macrophages, which facilitated the delivery of vancomycin to the intracellular bacteria, and that a single dose of vancomycin (from gelatin nanospheres) improved the survival of *S. aureus*-infected zebrafish larvae [65]. A similar approach was performed by Fenaroli et al. (2020) using pH-sensitive polymersomes as vesicles to target infected macrophages [64].

For the same purpose, namely of targeting intracellular *S. aureus*, Zhang et al. (2018) have studied a photochemical internalization (PCI) method in zebrafish larvae to enhance the intracellular activity of gentamicin. Infection of 30 hpf larvae was performed systemically via microinjection in either the posterior blood island or the Duct of Cuvier. At 2 hpi, larvae were treated via microinjection with gentamicin alone or combined with a photosensitizer, and 2 h later the larvae were illuminated for 10 min. The photosensitizer integrates into membranes of endocytic vesicles, which harbor and hinder gentamicin from reaching intracellular *S. aureus*. Upon illumination, photosensitizer-bound membranes are disrupted and release the drug, thus enabling the killing of bacteria. Survival analysis of zebrafish larvae revealed that 0.1 ng gentamicin delivered by photochemical internalization is equally efficient as 0.4 ng gentamicin alone. With this method, they showed that in vivo photochemical-induced release of gentamicin improves its in vivo activity, and that this delivery technique is able to lower the required doses for eradicating intracellular bacteria [66].

The zebrafish larvae model provides additional and important advantages compared to the invertebrate models mentioned in Chapter 2 that are used in early drug discovery. Despite being employed for studying the antibacterial effect of compounds and drugs in a variety of different setups, they can be easily combined with other established zebrafish larvae models. In the field of antibiotic research, it is already highly important at the discovery stages to assess the toxicity and in vivo stability of new potential drugs. Typically, such studies are performed using mouse models but, in recent years, it became apparent that the zebrafish larvae are equally predictive in the context of organ-specific toxicity and metabolism of drugs.

Compared to cell-based in vitro assays, zebrafish possess functional organs that develop within a few days post fertilization such as the heart, liver, kidneys and blood–brain barrier. Thus, it provides an early insight into absorption, distribution, metabolism, excretion and toxicity (ADMET) parameters [65]. Most zebrafish organs perform the same functions as their human counterparts, as well as hematopoietic system and cardiovascular physiology. Recent studies showed a 71% protein (and 82% disease-causing human protein) homology between human and zebrafish [41].

Several recent examples from the literature demonstrate the usefulness and predictivity of zebrafish larvae for studying ADMET properties of drugs and early-stage compounds. Cornet et al. (2017) have developed an assay named ‘ZeGlobalTox’ where they used transgenic zebrafish larvae expressing different fluorescent proteins to study the organ-specific toxicity of several drugs. In addition to the assessment of acute toxicity and developmental anomalies, they aimed to study the drug toxicity on organ physiology and function. Zebrafish larvae at 96 hpf were incubated separately at 28 °C with twenty-four different tested drugs including positive controls. Cardiotoxicity, neurotoxicity and hepatotoxicity were assessed at 100, 120 and 132 hpf. They reported the ZeGlobalTox assay as a reliable, sensitive and accurate tool to check specific organ toxicities and to red flag toxic compounds [68]. Diekmann and Hill (2012) have investigated, in their paper entitled “ADMETox in zebrafish”, the usage of mass spectroscopy for toxicity assessment in zebrafish larvae. They used zebrafish larvae for drug screening and to replicate their findings in rodents and humans [69]. Using Liquid Chromatography-High Resolution Mass Spectrometry (LC-HRMS), Park et al. (2020) have explored the metabolism of synthetic cannabinoids in zebrafish larvae after injection in the heart ventricle, caudal vein, yolk sac and the hindbrain, and compared them to those found in human urine. As a result, it could be shown that zebrafish larvae produce an authentic spectrum of human metabolites. They also assessed the spatial drug distribution of the compound and its metabolites by mass spectrometry imaging (MSI) in treated zebrafish larvae. Their findings presented an additional insight related to the study of metabolites in zebrafish larvae upon drug injection in different sites [70].

Taken together, zebrafish larvae represent an evolving model to study drug pharmacokinetics, and the in vivo efficacy and safety of early-stage compounds and drugs. Importantly, this model can be easily used in early drug discovery helping to select promising new compounds. By this, new compounds exhibiting, e.g., toxicity issues or rapid clearance can be excluded at an early stage, thus reducing the number of required animal experiments. Nevertheless, there are several challenges and limitations for the use of zebrafish, some of which might be overcome by further method improvements. The most critical aspect when using zebrafish larvae as a drug screening tool is the prediction of exposure concentrations, which would be needed to inform dosing schemes in, e.g., rodent models. It is not easy to quantify drug exposures, especially the amount of drug that will be absorbed when soaking the larvae. This is due to the fact that compounds can be taken up by the larvae either passively through their skin or, at later developmental stages, actively through the gills, and orally. Thus, there is no direct correlation between compound concentration in the medium and the in vivo concentration (total amount per larva). Moreover, many early-stage compounds display insufficient aqueous solubility, which can lead to compound precipitation in the fish water and, thus, incorrect assumptions regarding available doses. These issues can be overcome by injecting test compounds into the zebrafish larvae, but, at the same time, this will limit the usage of the larvae as a high-throughput screening tool. Moreover, in mammalian models, drug levels are often measured directly in serum or tissues, yet such measurements are more challenging in zebrafish due to the small size of larvae. The advancement of MSI can help to overcome some of these hurdles in order to conclude on drug distribution in the larvae. The anatomical and physiological differences between zebrafish and mammals present an additional challenge and, finally, the ability of zebrafish to regenerate multiple tissues has an impact on the predictiveness of toxicological studies [55].

## 5. Conclusion

In this review, available zebrafish larvae models of *S. aureus* infection are summarized, and current examples from the literature, with a focus on infection biology and drug discovery, are highlighted. Further, advantages of zebrafish larvae over invertebrate models and some limitations are discussed. Ultimately, zebrafish larvae are presented as an evolving in vivo model in (antibiotic) drug discovery, which is currently being used to study the efficacy, safety, and metabolism of biologically active compounds.

## Figures and Tables

**Table 1:** Reported studies of zebrafish larvae models to investigate *Staphylococcus aureus* disease biology. PC: pericardial cavity; 4V: fourth hindbrain ventricle; YCV: yolk circulation valley; DC: Duct of Cuvier; CV: caudal vein; PCV: posterior cardinal vein; YB: yolk body; dpf: days post fertilization; hpf: hours post fertilization; hpi: hours post infection.

Aim	Infection Route	Approach	Outcome	Reference
Study of pathogenesis	PC, Eye, 4V, YCV, DC, CV, YB	injection of various doses of *S. aureus* into different sites of zebrafish larvae at 36 hpf	analysis of survival, bacterial proliferation and myeloid cell phagocytosis	[43]
	YCV	co-infection with two *S. aureus* strains at 30 or 54 hpf, generation of phagocyte-depleted larvae using the morpholino method	analysis of survival, bacterial strain ratios and myeloid cell phagocytosis	[44]
	YCV	injection of *S. aureus* at 1 or 2 dpf, knockdown of *sqstm1* using the morpholino method	analysis of survival, myeloid cell phagocytosis and recruitment of autophagy receptors to *S. aureus*	[35]
	YCV	co-injection of *S. aureus* combined with a virulence-attenuated mutant as well as *S. aureus* and *M. luteus* at 30 hpf	analysis of survival and bacterial proliferation	[45]
	YCV	injection of *S. aureus* at 30 hpf, generation of neutrophil-enriched (*irf8* knockdown) as well as NADPH oxidase function deprived embryos using the morpholino method, CRISPR-mediated knockdown of *atg5* and *atg16l1*	analysis of survival, host–pathogen interactions and recruitment of autophagosomal markers to *S. aureus*	[33]
	YCV	injection of 1:1:1 mixtures of erythromycin-, kanamycin- and teracycline-resistant variants of different *S. aureus* strains at 30 hpf	analysis of bacterial strain ratios	[46]
Study of antibiotic intervention on staphylococcal infection dynamics	YCV	injection of 1:1:1 mixtures of erythromycin-, kanamycin- and tetracycline-resistant variants of different *S. aureus* strains at 30 hpf, antibiotic treatment via water immersion, generation of phagocyte-depleted larvae using the morpholino method	analysis of bacterial strain ratios	[47]
Study of pathogenesis; identification of virulence factors	YCV	injection of *S. aureus* wild-type as well as mutants, generation of phagocyte-depleted larvae using the morpholino method	analysis of survival and bacterial proliferation	[48,49]
Study of host innate immunity	YCV, PCV	injection of *S. aureus* at 2 dpf. knockdown of *14-3-3ζ* using the morpholino method	analysis of survival and myeloid cell phagocytosis	[50]
	YCV	injection of *S. aureus* at 30 hpf, knockdown of *trkA* using the morpholino method	analysis of survival, bacterial proliferation and neutrophil migration	[51]
Study of virulence	4V	injection of *S. aureus* wild-type and mutants as well as co-injection of different strains at 3 dpf	analysis of survival, bacterial proliferation, recruitment of immune cells and cytokine response using qRT-PCR	[34]
	PC	injection of *S. aureus* wild-type as well as mutant at 30–32 hpf	analysis of survival and bacterial proliferation	[52]
	YCV	injection of different *S. aureus* strains at 30 hpf	analysis of survival	[53]
Study of virulence and cross-resistance	DC	injection of *S. aureus* at 48 hpf, knockdown of hepcidin using the morpholino method	analysis of survival	[54]

**Table 2:** Reported studies of zebrafish larvae models in *Staphylococcus aureus* drug discovery. YCV: yolk circulation valley; CV: caudal vein; YB: yolk body; PC: pericardial cavity; 4V: fourth hindbrain ventricle; DC: Duct of Cuvier; PCV: posterior cardinal vein; dpf: days post fertilization; hpf: hours post fertilization; hpi: hours post infection.

Aim	Infection route	Approach	Outcome	Reference
Study of toxicity and efficacy of new antimicrobials	YCV	assessment of lethality, developmental toxicity and cardiotoxicity at 6 hpf, assessment of hepatotoxicity at 72 hpf, injection of MRSA at 30 hpf and treatment with antibacterials at 2 hpi	phenotypic assessment, analysis of survival, fluorescence intensity and bacterial proliferation	[58]
	bath water immersion	assessment of cardiotoxicity at 3 dpf, infection of 2 dpf larvae via bath water exposure to different concentrations of MRSA, treatment along with infection via bath water immersion	phenotypic assessment, analysis of survival	[59]
	CV, bath water immersion	assessment of acute toxicity at 2 hpf, infection of larvae with *S*. *aureus* either via microinjection into the CV or bath water immersion, treatment of larvae along with infection	phenotypic assessment, analysis of survival and bacterial proliferation, histopathological analysis	[60]
Study of efficacy of new antimicrobials	YCV, YB	injection of MRSA or MRSA grown in Epicatechin gallate at 30 hpf, treatment via bath water immersion	analysis of survival and NADPH-oxidase dependent respiratory burst	[61]
Study of efficacy using new antibiotic delivery systems	PC, 4V	injection of MRSA at 48 hpf, treatment via injection into the PCV 1 hpi	analysis of survival, fluorescence intensities and delivery of the drug to macrophages	[62]
	YCV, 4V	injection of *S. aureus* at 30 hpf, treatment with free Clarithromycin or encapsulated in PLGA nanocapsules via bath water immersion 2 hpi	analysis of survival and bacterial proliferation	[63]
	PCV	injection of *S. aureus* at 2 dpf, treatment with free drugs or drug loaded polymersomes via injection into the PCV at 20 hpi	analysis of bacterial proliferation, biodistribution of polymersomes and delivery of drugs to macrophages	[64]
	CV, DC, tail muscle	injection of *S. aureus* at 3 dpf into the DC and the tail muscle to study biodistribution and internalization of nanospheres, injection of *S. aureus* at 30 hpf into the CV, treatment with free Vancomycin or Vancomycin loaded gelatin nanospheres via CV injection at 2 hpi	analysis of survival, biodistribution and internalization of the nanospheres into macrophages	[65]
	CV, DC	injection of *S. aureus* at 30 hpf treatment with Gentamicin alone or combined with a photosensitizer at 2 hpi, 10 min illumination at 2 hpt	analysis of survival and interaction of *S. aureus* with macrophages	[66]

## Data Availability

Not applicable.
